# Fatty Acids Regulate Porcine Reproductive and Respiratory Syndrome Virus Infection via the AMPK-ACC1 Signaling Pathway

**DOI:** 10.3390/v11121145

**Published:** 2019-12-10

**Authors:** Siwen Long, Yanrong Zhou, Dongcheng Bai, Wanjun Hao, Bohan Zheng, Shaobo Xiao, Liurong Fang

**Affiliations:** 1 State Key Laboratory of Agricultural Microbiology, College of Veterinary Medicine, Huazhong Agricultural University, Wuhan 430070, China; lsw1624@hotmail.com (S.L.); zhengbohan@webmail.hzau.edu.cn (B.Z.); vet@mail.hzau.edu.cn (S.X.); 2 The Key Laboratory of Preventive Veterinary Medicine in Hubei Province, Cooperative Innovation Center for Sustainable Pig Production, Wuhan 430070, China; 3 Laboratory of Animal Virology, College of Veterinary Medicine, Huazhong Agricultural University, 1 Shi-zi-shan Street, Wuhan 430070, Hubei, China

**Keywords:** porcine reproductive and respiratory syndrome virus (PRRSV), fatty acid, AMP-activated protein kinase (AMPK), acetyl-CoA carboxylase 1 (ACC1)

## Abstract

Lipids play a crucial role in the replication of porcine reproductive and respiratory syndrome virus (PRRSV), a porcine virus that is endemic throughout the world. However, little is known about the effect of fatty acids (FAs), a type of vital lipid, on PRRSV infection. In this study, we found that treatment with a FA biosynthetic inhibitor significantly inhibited PRRSV propagation, indicating the necessity of FAs for optimal replication of PRRSV. Further study revealed that 5′-adenosine monophosphate (AMP)-activated protein kinase (AMPK), a key kinase antagonizing FA biosynthesis, was strongly activated by PRRSV and the pharmacological activator of AMPK exhibited anti-PRRSV activity. Additionally, we found that acetyl-CoA carboxylase 1 (ACC1), the first rate-limiting enzyme in the FA biosynthesis pathway, was phosphorylated (inactive form) by PRRSV-activated AMPK, and active ACC1 was required for PRRSV proliferation, suggesting that the PRRSV infection induced the activation of the AMPK–ACC1 pathway, which was not conducive to PRRSV replication. This work provides new evidence about the mechanisms involved in host lipid metabolism during PRRSV infection and identifies novel potential antiviral targets for PRRSV.

## 1. Introduction

Porcine reproductive and respiratory syndrome (PRRS) has been one of the most economically prominent swine diseases worldwide for decades [1]. It is typically characterized by reproductive failure in pregnant sows, reduced boar semen quality, and severe respiratory disease in infected newborn and young pigs [2]. PRRS virus (PRRSV), the etiologic agent of PRRS, is an enveloped, positive sense, single-stranded RNA virus classified within the order *Nidovirales* in the family *Arteriviridae* [3]. Unfortunately, the current commercial vaccines for PRRS fail to provide sustainable disease control due to the immunosuppression and genetic heterogeneities of PRRSV, and no efficient antiviral agents against PRRS are available presently, which leads to globally rising outbreaks of PRRS and subsequent tremendous economic losses [4,5,6]. The development of potent broad-spectrum antiviral therapy against PRRS, by better understanding the pathogenesis of the disease, is essential to reduce the transmission of PRRS [7].

Viruses always exploit and reprogram cellular components to form an optimal environment for the replication of viral progenies, many of which are dependent on cellular lipid signaling, synthesis, and metabolism [8,9,10]. The close interaction between virus and host cellular lipids occurs at several stages in the virus replication cycle, including replication, assembly, and secretion [11]. As more is learned about the role of lipids in virus replication, the reprogramming of cellular lipid metabolic pathways under virus infection, such as glycolytic pathway and cholesterol and fatty acid (FA) synthesis signaling, will be a rapidly emerging theme. For example, Dengue virus (DENV) provokes a remarkable increase in intracellular cholesterol and FA levels and stimulates glycolysis for optimal replication [12,13,14]. Accordingly, pharmacological inhibitors targeting lipid metabolic pathways involved in the viral replication cycle provide novel targets for future antiviral agent development. The drugs that disrupt FA biosynthesis pathways have been reported to possess an antiviral effect against multiple enveloped viruses, including hepatitis delta virus, hepatitis C virus (HCV), human immunodeficiency virus, Rift Valley fever virus, and Hepatitis B virus [15,16,17,18,19], confirming the significance of FAs in virus replication.

5′-adenosine monophosphate (AMP)-activated protein kinase (AMPK), a heterotrimeric complex consisting of a catalytic alpha subunit and regulatory beta and gamma subunits, is an evolutionarily conserved serine/threonine kinase [20]. The first known and most important function of AMPK is the regulation of lipid metabolism. AMPK is activated through phosphorylation of the threonine (Thr) residue 172 on the alpha subunit, which inhibits both FAs and cholesterol synthesis, mainly by separately inducing the phosphorylation of their key rate-limiting enzymes, acetyl-coA carboxylase 1 (ACC1) and HMG-CoA Reductase (HMGCR) [21,22]. Additionally, AMPK plays a significant role in maintaining dynamic energy homeostasis [23]. Intensive studies spanning decades have demonstrated that AMPK is closely linked with multiple metabolic pathways and physiological processes. An imbalance in AMPK activity is associated with various chronic diseases including metabolic syndrome, obesity, stress, type II diabetes, or even reduced longevity and the promotion of cancer [24,25,26,27]. Because of its significance, AMPK has been considered a potential target in the treatment of multiple diseases.

In the work described here, we demonstrated that the pharmacological inhibitor (C75) of the FA synthesis pathway can suppress PRRSV infection, suggesting a significant role of FAs during PRRSV infection. Furthermore, we found that the AMPK activity was positively regulated in PRRSV-infected cells and PRRSV-activated AMPK drove a decline of ACC1 activity in turn. Both pharmacological activators of AMPK and inhibitors of ACC1 had anti-PRRSV effects, indicating that host cells antagonized PRRSV infection via activating the AMPK-ACC1 signaling pathway. These findings highlight FA metabolism as a new potential antiviral target.

## 2. Materials and Methods

### 2.1. Cells, Virus, and Reagents

PK-15^CD163^ cells (gifted by En-min Zhou at Northwest A&F University, China) [28], a pig kidney cell line stably expressing the PRRSV receptor CD163, were cultured in Dulbecco’s Modified Eagle’s Medium (DMEM) (Invitrogen, CA USA). Primary porcine alveolar macrophages (PAMs) were kept in Roswell Park Memorial Institute (RPMI)-1640 medium (HyClone, Utah, USA). PRRSV strain WUH3, a highly pathogenic type 2 (North American) PRRSV, was isolated from the brains of pigs suffering from high-fever syndrome in China [29]. PRRSV was amplified, and the titer was determined in PK-15^CD163^ cells.

The FA synthase inhibitor C75 (C5490) was purchased from Sigma (MA, USA), dissolved in DMSO at a concentration of 15 mM, and stored at −80 °C. The AMPK activator A769662 (HY-50662) and ACC1 inhibitor CP-640186 (HY-15259) were obtained from MedChemExpress (MCE, NJ, USA) and stored at −80 °C at the concentration of 150 mM and 5 mM, respectively.

### 2.2. Small Interfering RNAs (siRNAs) and Transfection

The siRNAs targeting porcine AMPK or negative control (NC) siRNA were designed by Genepharma (Suzhou, China), and the sequences are listed in Table 1. The siRNAs were dissolved in DEPC water and transfected at a final concentration of 50 nM using jetPRIME® Transfection Reagent (Polyplus-transfection^®^ SA, Strasbourg, France).

### 2.3. Cytotoxicity Assay

Different concentrations of C75, A769662, or CP-640186 were added to PK-15^CD163^ cells, or PAMs, and incubated for 30 h. Cell viability was then determined using the 3-(4,5-dimethyl-2-thiazolyl)-2,5 diphenyl-2H-tetrazoliumbromide (MTT) assay.

### 2.4. Western Blot Assay

PK-15^CD163^ cells cultured in 6-well plates were rinsed with phosphate-buffered saline (PBS) three times, followed by the addition of 200 µL/well of lysis buffer supplemented with protease inhibitor (PMSF) and phosphatase inhibitor (Beyotime, Shanghai, China). Each sample was collected and denatured in 5× SDS loading buffer by boiling at 95 °C for 10 min for sodium dodecyl sulfate polyacrylamide gel electrophoresis (SDS-PAGE). Then, proteins were electroblotted onto PVDF membranes (Millipore, Darmstadt, Germany). Antibodies against AMPK (A1229), ACC1 (A15606), p-ACC1 (AP0298), and β-actin (AC026) were obtained from Abclonal Technology (Wuhan, China). Antibodies against p-AMPK (#2535) were purchased from Cell Signaling Technology (CST, MA, USA). Monoclonal antibody against PRRSV N protein was made in our laboratory. The ratios of p-AMPK/AMPK, p-ACC1/ACC1, and PRRSV-N/β-actin were analyzed using Image J Software (National Institutes of Health, Bethesda, MD, USA).

### 2.5. Indirect Immunofluorescence Assay

Cells seeded in 24-well plates were fixed with 4% paraformaldehyde (precooled at 4 °C) for 10 min and then immediately permeabilized with methanol (precooled at −30 °C) for 15 min prior to the addition of blocking reagent (5% BSA). Next, cells were incubated with the primary antibody against PRRSV-N protein for 1 h and washed three times with PBS, followed by incubation with Alexa Fluor 594-conjugated donkey anti-mouse IgG (Jackson ImmunoResearch, PA, USA). Cell nuclei were stained with 4′,6-diamidino-2-phenylindole (DAPI; Beyotime, Shanghai, China) for 45 min. Fluorescent images were acquired with Axio-Imager_LSM-800 (Zeiss, Germany).

### 2.6. TCID_50_ Assay

PK-15^CD163^ cells were pretreated with DMSO or inhibitors for 6 h and then infected with PRRSV (MOI = 0.5) in the presence of inhibitors at corresponding concentration for 24 h. PRRSV samples were harvested through repeated freezing–thawing and centrifugation for TCID_50_ assay. Next, fresh PK-15^CD163^ cells were seeded in 96-well plates and then infected with serial 10-fold dilutions of PRRSV samples in eight replicates. The plates were incubated for approximately 96 h before virus titers were calculated. PRRSV titers were expressed as TCID_50_ per milliliter using the Reed–Muench method. 

### 2.7. Plaque Assay

All the samples were harvested as described in 2.6. PK-15^CD163^ cells seeded in six-well plates were infected with serial 10-fold dilutions of PRRSV samples for 2 h, the viral inoculums were removed, and cells were overlaid with a mixture containing half 1.6% low-melting-point agarose and half 2% DMEM. These were incubated at 4 °C for 10 min before being cultured at 37 °C for approximately 48 h. Finally, cells were stained with neutral red and the number of plaques was counted.

### 2.8. Statistical Analysis

GraphPad Prism 5 software (GraphPad Software, CA, USA) was used for data analysis using two-tailed unpaired *t* tests.

### 2.9. RNA Extraction and Quantitative Real-Time PCR (qRT-PCR)

Total RNA was extracted with TRIzol reagent (Invitrogen, CA, USA) and then reverse transcribed into cDNA by reverse transcriptase (Roche, Mannheim, Germany). The qRT-PCR experiments were performed in triplicate. Absolute quantitative mRNA levels of PRRSV *NSP9* gene (5′-GTTGATGGTGGTGTTGTGCT-3′ and 5′-AGACCAATTTTAGGCGCGTC-3′) were calculated using its standard plasmid’s amplification curve. Real-time PCR was performed using Power SYBR green PCR master mix (Applied Biosystems, CA, USA) in an ABI 7500 real-time PCR system (Applied Biosystems, CA, USA).

## 3. Results

### 3.1. Blocking Fatty Acid Synthesis Inhibits PRRSV Replication

**Figure 1:**
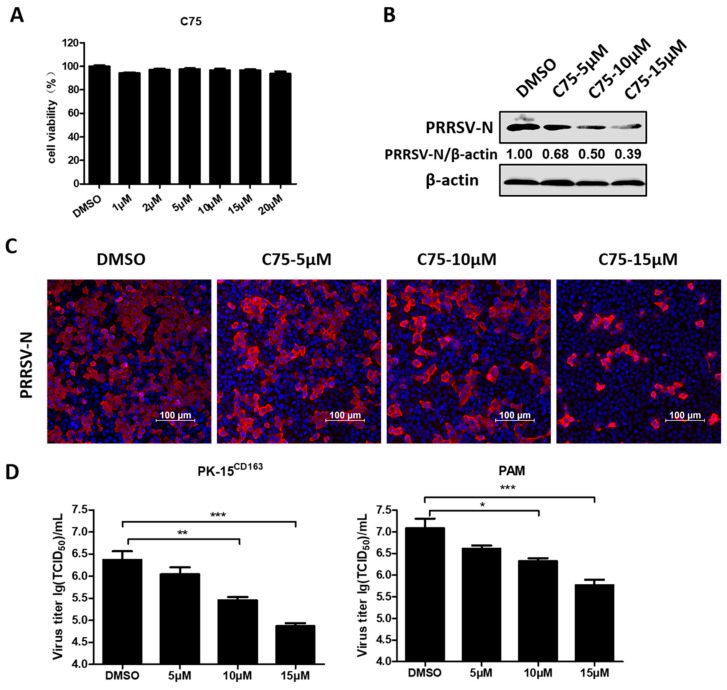
The pharmacological inhibitor (C75) inhibited porcine reproductive and respiratory syndrome virus (PRRSV) replication. (**A**) PK-15^CD163^ cells were incubated with various concentrations of C75 or DMSO, as a control, for 30 h for the 3-(4,5-dimethyl-2-thiazolyl)-2,5 diphenyl-2H-tetrazoliumbromide (MTT) assay to determinate the cytotoxicity of C75. (**B**) PK-15^CD163^ cells were pretreated with C75 at the indicated concentrations (5, 10, and 15 µM) for 6 h. The cells were then infected with PRRSV (MOI = 0.5) in the presence of indicated concentrations of C75 and harvested at 24 h post-infection (hpi) for western blot assay, using a specific antibody against PRRSV-N protein. β-actin was used as a loading control. (**C**,**D**) Indirect immunofluorescence assay (PRRSV-N, red; nuclei, blue) (**C**) and TCID_50_ assay (**D**) were separately performed to determine the expression levels of PRRSV-N protein, virus titers in PK-15^CD163^, or porcine alveolar macrophages (PAMs) treated with C75 as described in (**B**). Data are expressed as means and standard deviations from three independent experiments. *, 0.01 ≤ *p* < 0.05; **, 0.001 ≤ *p* < 0.01; ***, *p* < 0.001.

To investigate the role of FAs in PRRSV infection, C75, a FA inhibitor targeting FA synthase (FASN) was used. First, a cell cytotoxicity assay was tested in PK-15^CD163^ cells and concentrations below 20 µM of C75 were chosen for further experiments (Figure 1A). The results of the western blot assays revealed that treatment with C75 significantly inhibited PRRSV-N production in a dose-dependent manner, as seen in Figure 1B. Moreover, we observed a notable decrease in the fluorescence intensity of PRRSV-N protein and virus titer in the presence of C75 through the indirect immunofluorescence assay and TCID_50_ assay, as seen in Figure 1C and 1D. These confirmed the anti-PRRSV activity of C75, indicating that FAs are important players during PRRSV infection.

### 3.2. PRRSV Infection Upregulates AMPK Activity

**Figure 2:**
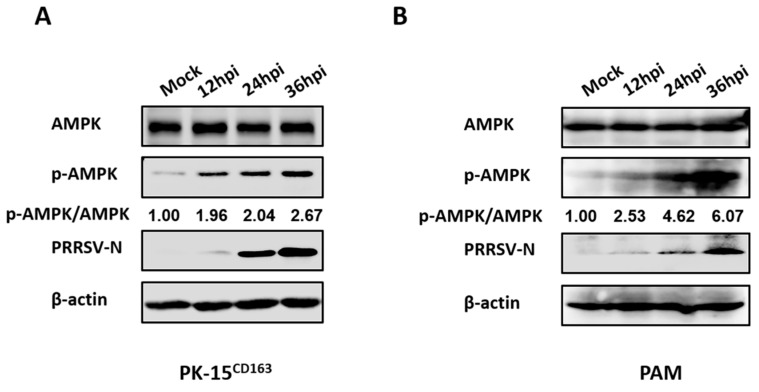
PRRSV infection upregulated AMPK phosphorylation levels. (**A**) PK-15^CD163^ or (**B**) PAMs were infected with PRRSV (MOI = 0.5) for 12, 24 or 36 h. Lysates were collected and analyzed by western blot assay for the levels of total AMPK, phosphorylated-AMPK (p-AMPK) and PRRSV-N protein. β-actin was measured as a loading control.

FAs are required for optimal replication of PRRSV, which prompted us to further investigate whether intracellular FA biosynthesis signaling pathways are affected by PRRSV. Given the importance of AMPK as a key kinase in FA metabolism, AMPK activity variation during PRRSV infection was evaluated through detection of the phosphorylation levels of AMPK at residue Thr172 of the alpha subunit [30]. Only minor differences in total protein levels of AMPK were observed, whereas AMPK phosphorylation levels rose in a time-dependent manner with a 2.6-fold increase in PK-15^CD163^ cells, as seen in Figure 2A, and a 6.0-fold increase in PAMs, as seen in Figure 2B at 36 h post-infection (hpi) in the PRRSV-infected group compared with the mock-infected group. These results confirmed that PRRSV infection strongly activated AMPK signaling.

### 3.3. AMPK Activation Restricts PRRSV Infection

**Figure 3:**
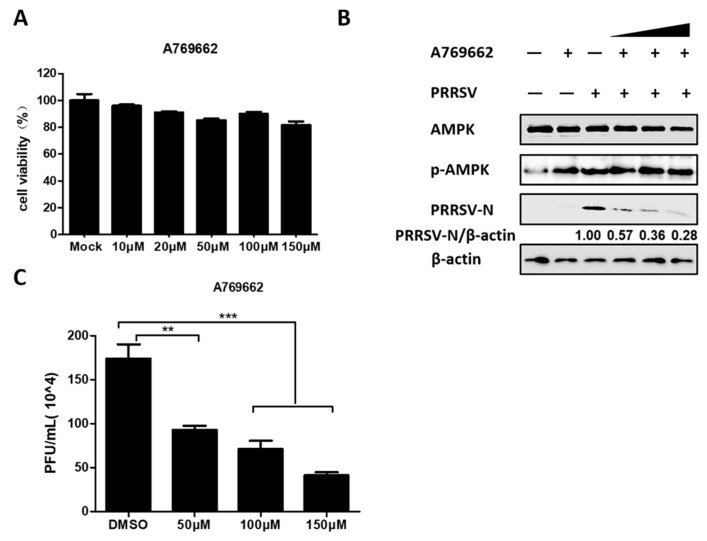
Active AMPK antagonized PRRSV infection. (**A**) PK-15^CD163^ cells were incubated with different concentrations of A769662 or DMSO, as a control for 30 h for MTT assay. (**B**,**C**) The effect of A769662 (50, 100, and 150 µM) on PRRSV infection (MOI = 0.5) was evaluated at 24 hpi through western blot assay detecting protein expression levels of PRRSV-N, p-AMPK, total AMPK, and β-actin (**B**), and plaque assay evaluating virus titers (**C**). The results represent the means and standard deviations from three independent experiments. **, 0.001 ≤ *p* < 0.01; ***, *p* < 0.001.

The above data demonstrated that PRRSV significantly activated AMPK, so we continued to investigate whether AMPK activation affects PRRSV replication. AMPK activator A769662 was selected for further research. First, the cytotoxicity of A769662 was evaluated with the MTT assay, which revealed that no obvious cytotoxicity was observed in PK-15^CD163^ cells treated with A769662 at concentrations below 150 µM, as seen in Figure 3A. Next, PRRSV-infected PK-15^CD163^ cells were treated with various concentrations of A769662. The results of western blot assay indicated that A769662 reduced PRRSV-N expression in a dose-dependent manner, as seen in Figure 3B. Likewise, the results of the plaque assay revealed an approximately four-fold decline in the number of plaques formed in A769662-treated cells compared with DMSO-treated cells, as seen in Figure 3C. Altogether, these data indicated that activated-AMPK had an antiviral effect on PRRSV in PK-15^CD163^ cells.

**Figure 4:**
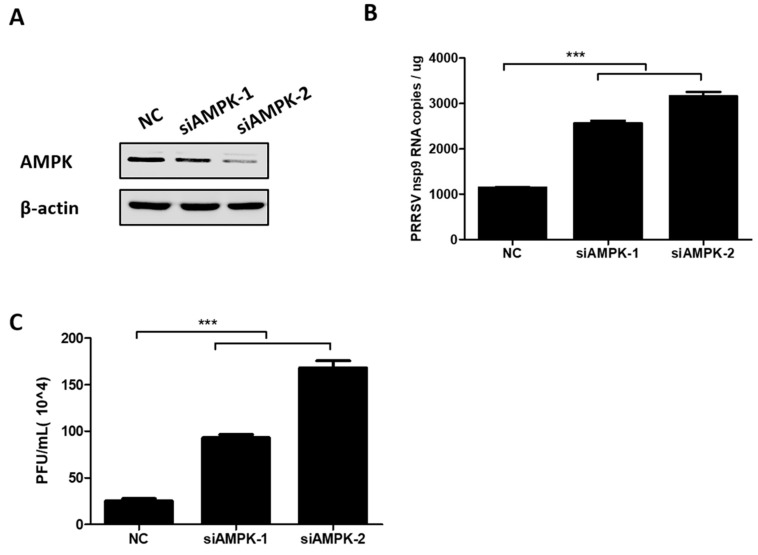
Knockdown of AMPK-enhanced PRRSV infection. (**A**) PK-15^CD163^ cells were transfected with AMPK-specific siRNAs (siAMPKs) or NC siRNA. At 36 h post transfection, cells were harvested to determine the knockdown efficiency through western blot assay. (**B**,**C**) PK-15^CD163^ were transfected with siAMPKs or NC siRNA. After 24 h, cells were infected with PRRSV (MOI of 0.5) and processed to detect the mRNA levels of the PRRSV *NSP9* gene by qRT-PCR assay (**B**) and virus titers by plaque assay (**C**) at 24 hpi. The results represent the means and standard deviations from three independent experiments. ***, *p* < 0.001.

To further evaluate the effect of AMPK on PRRSV infection, two AMPK-specific siRNAs (siAMPK-1 and siAMPK-2) were designed, and the interference efficiency of each siRNA was determined by western blot assay. The results show that both siRNAs were able to knockdown AMPK expression, and siAMPK-2 displayed higher knockdown efficiency, as seen in Figure 4A. In PK-15^CD163^ cells transfected with these two siRNAs, PRRSV mRNA levels and virus titers were examined by qRT-PCR assay and plaque assay, respectively. As shown in Figure 4B,C, knockdown of AMPK promoted the RNA levels of the PRRSV *NSP9* gene and increased PRRSV titers. Moreover, siAMPK-2 had better silence efficiency and was more favorable for PRRSV infection. All the above results suggest that PRRSV-induced AMPK activation is not conducive to PRRSV.

### 3.4. AMPK is Involved in PRRSV-Mediated ACC1 Activity Reduction

**Figure 5:**
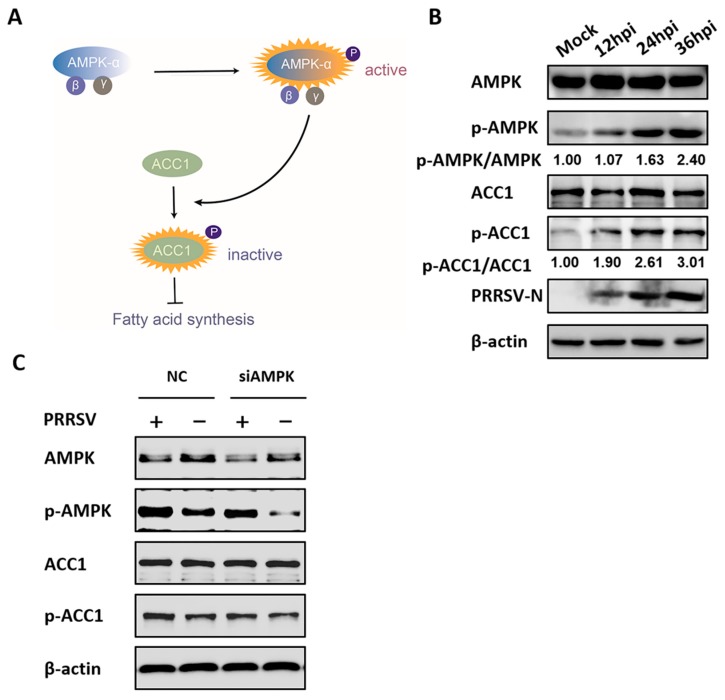
Acetyl-CoA carboxylase 1 (ACC1) activity is regulated by AMPK during PRRSV infection. (**A**) Model of fatty acid (FA) synthesis via the AMPK–ACC1 pathway. The levels of phosphorylated ACC1 (inactive form) were enhanced by active AMPK, resulting in inhibitory FA synthesis. (**B**) PK-15^CD163^ cells were infected with PRRSV (MOI = 0.5) for 12, 24, or 36 h. Lysates were collected and tested by western blot assay for the levels of total AMPK, p-AMPK, total ACC1, phosphorylated ACC1 (p-ACC1), and PRRSV-N protein. β-actin was measured as a loading control. (**C**) PK-15^CD163^ cells were transfected with AMPK-specific siRNAs or control siRNA (NC) for 24 h and infected with PRRSV (MOI = 0.5) for 24 h for western blot analysis.

Considering that AMPK inactivated acetyl-CoA carboxylase 1 (ACC1), the rate-limiting enzyme of FA synthesis, and suppressed the levels of cellular FAs, as seen in Figure 5A, the effect of PRRSV-activated AMPK on ACC1 activation in PRRSV-infected cells was tested to further investigate the mechanism by which AMPK reduces PRRSV replication. As shown in Figure 5B, the levels of phosphorylated ACC1 (inactivated ACC1) increased in a time-dependent manner after PRRSV infection, which was consistent with the activation status of AMPK. Furthermore, we found that specific siRNA-mediated knockdown of AMPK significantly decreased PRRSV-induced ACC1 phosphorylation, as seen in Figure 5C, suggesting that ACC1 activity is modulated by AMPK during PRRSV infection. This proved that the AMPK–ACC1 pathway, related to the negative regulation of FA metabolism, was activated in PRRSV-infected cells.

### 3.5. ACC1 Inhibitor Disrupts PRRSV Replication

**Figure 6:**
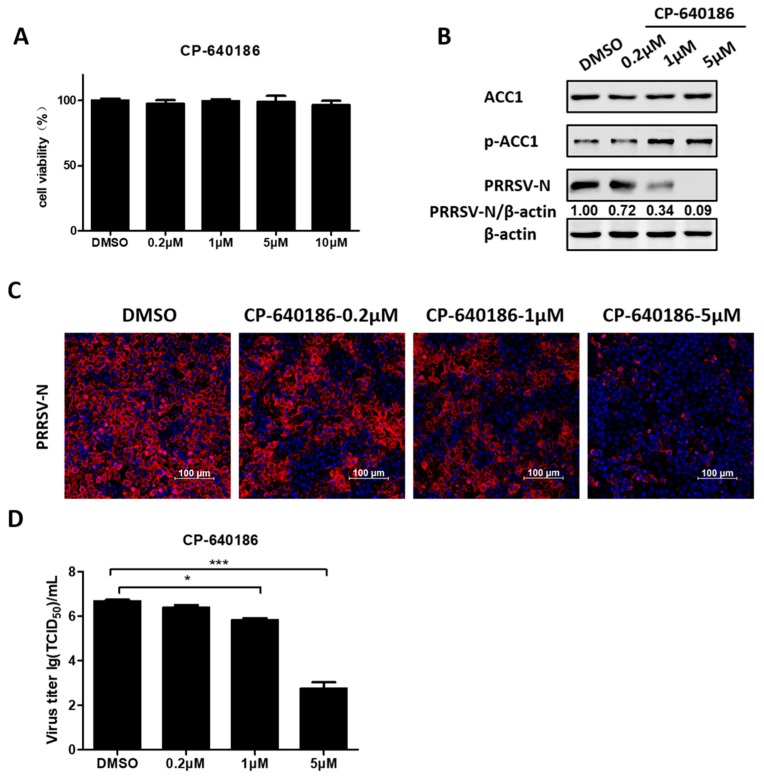
CP-640186 an ACC1-specific pharmacological inhibitor, inhibited PRRSV infection. (**A**) PK-15^CD163^ cells were incubated with various concentrations of CP-640186 or DMSO as a control for 30 h for MTT assay. (**B**) PK-15^CD163^ cells were pretreated with CP-640186 at various concentrations (0.2, 1, and 5 µM) for 6 h prior to PRRSV infection (MOI = 0.5). The infected cells were cultured in the presence of indicated concentrations of CP-640186 and harvested at 24 hpi for western blot analysis using a specific antibody against PRRSV-N protein, and β-actin was used as a loading control. (**C**) The distribution of PRRSV-N protein (red) and nuclei (blue) in PRRSV-infected PK-15^CD163^ cells was evaluated by confocal microscopy at 24 hpi. (**D**) Virus titers were detected by TCID_50_ assay. Data are expressed as means and standard deviations from three independent experiments. *, 0.01 ≤ *p* < 0.05; ***, *p* < 0.001.

To further assess the effect of ACC1 on PRRSV infection, PK-15^CD163^ cells were treated with CP-640186, an ACC1-specific pharmacological inhibitor. As shown in Figure 6A, no obvious cytotoxicity was observed after treatment with CP-640186 at concentrations below 10 µM. Next, PRRSV-infected PK-15^CD163^ cells were treated with various concentrations of CP-640186 (0.2, 1, and 5 µM). Western blot analysis showed that CP-640186 inhibited the expression of PRRSV-N protein in a dose-dependent manner, as seen in Figure 6B. Additionally, we observed a notable decrease in the fluorescence intensity of PRRSV-N protein and PRRSV titers in the presence of CP-640186 through indirect immunofluorescence assay and TCID_50_ assay, respectively, as seen in Figure 6C,D. These data are consistent with the above results that both C75 (FA synthase inhibitor) and A769663 (AMPK activator) restricted PRRSV proliferation, collectively emphasizing the significance of FAs in PRRSV infection.

## 4. Discussion

In recent years, we have gained insights into the importance of lipids in virus replication. For non-enveloped viruses, glycosphingolipids (GSLs) can directly function as attachment receptors to initiate infection [31]. For enveloped viruses, such as HCV and bovine viral diarrheal virus (BVDV), lipid traffic receptors have been described as indirect co-receptors for viral entry [32]. Furthermore, enveloped viruses can regulate lipid metabolism to rearrange intracellular membrane systems, mainly endoplasmic reticulum (ER), mitochondria, and Golgi, and to establish replication sites to concentrate viral and host proteins required for viral replication [33]. Additionally, lipids can recruit viral RNAs to separate them from innate immune sensors, which is a novel strategy for viruses to avoid the host antiviral immune response [34]. For PRRSV, our previous research and that of other groups demonstrated that cholesterol and lipid rafts are potent participants in the PRRSV replication cycle [35,36,37,38]. Here, we found that fatty acids, another important kind of lipid, are essential components for PRRSV infection. However, the mechanism(s) involved in the FA regulation during PRRSV proliferation remains unknown. Previous studies have found that alteration of FA properties can drastically affect the topological structure of cellular membranes [39,40], interfere with membrane fusion, and remodel the envelopment required for virus replication [41,42], these may be potential subjects for future mechanistic studies.

**Figure 7:**
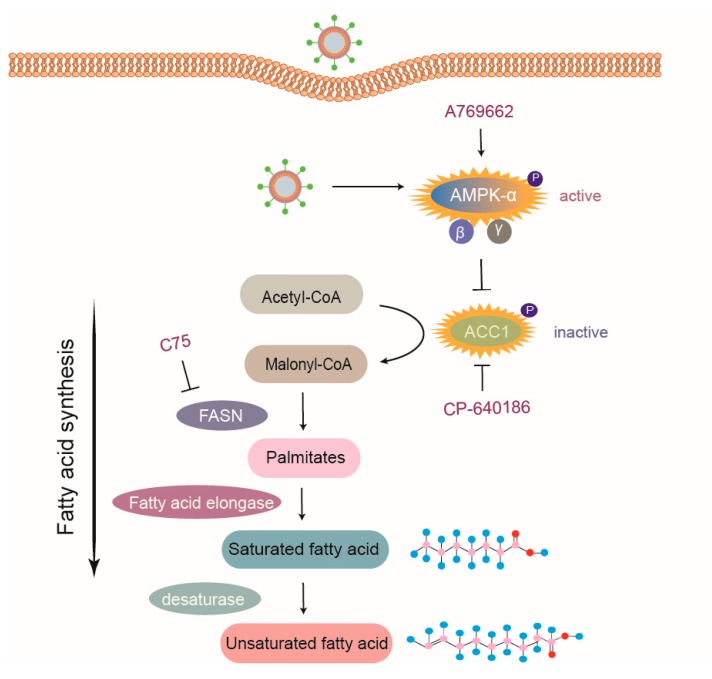
Model of PRRSV-mediated manipulation of the AMPK–ACC1 pathway. Acetyl-CoA is metabolized to malonyl-CoA via ACC1, which is then transformed into palmitates by FA synthase (FASN). Palmitates are prolonged through FA elongase to form saturated FAs, which are subsequently metabolized to unsaturated FAs by desaturase. PRRSV infection activated AMPK, thus resulting in an increased level of phosphorylated ACC1 (inactive form) to block FA synthesis. Pharmacological agents targeting various steps of FA synthesis pathway, such as C75 (inhibitor of FASN), CP-640186 (inhibitor of ACC1), and A769662 (activator of AMPK), suppressed FA biosynthesis and subsequently inhibited the replication of PRRSV. In summary, host cells limit PRRSV infection via the AMPK–ACC1 pathway.

FA synthesis is a process catalyzed by various enzymes; first, ACC1 catalyzes the transformation of acetyl-CoA into malonyl-CoA, which is then converted to de novo palmitate through FA synthase. Next, palmitate undergoes chain propagation to produce saturated FAs via FA elongase [43], as seen in Figure 7. It is common to suppress FA synthesis by inhibitors targeting related enzymes, which have been proven to decrease the replication of various viruses, including respiratory viruses [44] and flaviviruses [45]. Additionally, evidence has shown that drugs targeting ACC1 are effective for antiviral treatment [46,47,48]. In this study, the antiviral effects of ACC1 inhibitor (CP-640186) and FASN inhibitor (C75) on PRRSV replication were demonstrated as well, supporting the significance of studies on FA metabolism for the development of novel antiviral agents.

AMPK is a potent FA metabolic inhibitory regulator. Previous studies reported that some viruses inhibit AMPK activity to increase lipid deposition, which creates a conducive cellular lipidic environment for virus replication, such as Epstein–Barr virus, HCV, and DENV [49,50,51,52]. Conversely, human cytomegalovirus can activate AMPK to benefit its infection [53]. In this study, we found that PRRSV infection dramatically increased the levels of phosphorylated AMPK (active form), which antagonized PRRSV replication. Activated AMPK inhibited FA synthesis by reducing the activity of ACC1, the rate-limiting enzyme of FA biosynthesis pathways. Some viruses, such as Rift Valley fever virus, activate the AMPK–ACC1 pathway, leading to a decrease in FA levels and viral progenies [18]. Concomitant with that, enhanced ACC1 phosphorylation (inactive form) in PRRSV-infected cells was also shown to depend on AMPK activation, and the activation of the AMPK–ACC1 pathway resulted in suppressed PRRSV replication, as seen in Figure 7. However, FA synthesis is enhanced during infection with some other viruses through different mechanisms. For example, DENV NS3 protein interacts with FASN, which is then recruited to viral replication sites to upregulate FA production [13]. Moreover, human cytomegalovirus induces the expression of FA elongase to produce more saturated FA required for virus replication via mTOR and SREBP-1 pathways [54].

It is well known that viral infections are competitive processes between viruses and host cells. However, little is known about the molecular details of the influences of FAs on PRRSV replication. As an enveloped RNA virus, PRRSV might induce membrane expansion for entry and membrane rearrangement of ER to form a replication complex. This suggests that FAs, key components of cellular membranes, may be involved in the entry and replication stages in the PRRSV replication cycles. Moreover, for some viruses, such as HCV and enterovirus, virus proteins and RNA are transported to lipid droplets (LDs) as sites of virus assembly [55,56]. Considering that FAs are related to the formation of LDs, there is the possibility that FAs regulate PRRSV replication by affecting the production of LDs, which requires further research to confirm. In addition to the previously described regulatory functions, FA metabolism is involved in immune responses, such as inflammation signaling [57]. This information provides new opportunities for the study of potential associations between FA metabolism and PRRSV-induced clinical features, including interstitial pneumonia. Further investigation of the interactions between FAs and PRRSV infection is essential to improve our knowledge about the pathogenesis of PRRSV and provide important insights for the development of novel anti-PRRSV agents.

## 5. Conclusions

In conclusion, we found that PRRSV infection requires and scrambles FAs of host cells to produce progenies, while in response, host cells develop countermeasures to activate the AMPK–ACC1 signaling pathway involved in FA metabolism, thus suppressing PRRSV infection. These findings highlight FA metabolism as a new potential antiviral target against PRRSV.

## Figures and Tables

**Table 1:** The siRNA sequences targeting porcine 5′-adenosine monophosphate (AMP)-activated protein kinase (AMPK).

Gene Name	siRNA Sequence (Sense)	siRNA Sequence (Anti-Sense)
Si-AMPK-1	5′-GGUUCUCAGCUGCCUUUAUTT-3′	5′-AUAAAGGCAGCUGAGAACCTT-3′
Si-AMPK-2	5′-GCUUGCCAAAGGAAUGAUUTT-3′	5′-AAUCAUUCCUUUGGCAAGCTT-3′
NC	5′-UUCUCCGAACGUGUCACGUTT-3′	5′-ACGUGACACGUUCGGAGAATT-3′
